# Time-Resolved Four-Channel Jones Matrix Measurement of Birefringent Materials Using an Ultrafast Laser

**DOI:** 10.3390/ma15217813

**Published:** 2022-11-05

**Authors:** Zhenjia Cheng, Yuqin Zhang, Xuan Liu, Chengshan Guo, Changwei He, Guiyuan Liu, Hongsheng Song

**Affiliations:** 1 School of Science, Shandong Jianzhu University, Jinan 250101, China; 2 School of Physics and Electronics, Shandong Normal University, Jinan 250014, China

**Keywords:** birefringent materials, Jones matrix, ultrafast laser

## Abstract

A method for ultrafast time-resolved four-channel Jones matrix measurement of birefringent materials using an ultrafast laser is investigated. This facilitated the acquisition of a four-channel angular multiplexing hologram in a single shot. The Jones matrix information of a birefringent sample was retrieved from the spatial spectrum of a hologram. The feasibility of this approach was established by measuring the Jones matrix of starch granules in microfluidic chips and the complex amplitude distribution and phase delay distribution of liquid crystal cell at different voltages. Moreover, when the picosecond laser was switched to a femtosecond laser, ultrafast measurements were possible provided that the time interval between two detection pulses was larger than the pulse width.

## 1. Introduction

The polarization measurement of a light field is of increasing importance in optical analysis. This phenomenon is an important attribute of birefringent materials and can reflect structure, shape, refractive index, and other important properties [1]. Therefore, measurement of the polarization state of matter can facilitate the detection of cell and tissue pathologies [2,3,4,5,6], metallic structures [7,8], the estimation of the concentration of solutions [9,10,11,12], etc. Polarization measurement has been widely applied in biomedical science, material science, biophysics, and physicochemistry [13,14,15,16,17,18]. Although the polarization phenomenon of birefringent materials has many practical applications, the measurement of this phenomenon is typically challenging. Many methods have been proposed to address this challenge over the past few decades. Quantitative phase imaging [19,20] and digital holographic microscopy [21] are two established approaches for obtaining complex amplitude distributions. In 2008, Wang et al. proposed Jones phase microscopic imaging [22]. They used off-axis holography to realize the direct measurement of the 2D Jones matrix parameters of a sample for the first time. However, this technology is not suitable for dynamic research involving birefringent samples because the measurement of the Jones matrix parameters requires four steps. In addition, the rotation of the input and output polarizers must be precisely controlled during the measurement process. In 2017, Han et al. proposed a common-path off-axis digital holography polarization method [23], and in 2018, Alonso et al. proposed a dual-channel common-path interferometry technique [24]. Both methods require two-step measurements of samples to obtain complete polarization information. As such, they are only applicable to the study of static birefringent materials. In 2014, Liu et al. reported on a one-step Jones matrix polarization holography approach [25,26]. This method can extract the Jones matrix information of birefringent materials in a single measurement. However, two incoherent continuous light sources are required. Therefore, it is only possible to realize the measurement of general dynamic birefringent samples and does not facilitate ultrafast dynamic polarization measurements of samples. Kim et al. proposed a polarization holographic microscopy technique to extract the spatially resolved Jones matrix from anisotropic samples [21]. Several methods are available for acquiring polarization information from individual microscopic samples [27,28,29,30,31]. These methods all require continuous lasers, and few of them are suited for application to ultrafast measurement.

Ultrafast lasers (including picosecond and femtosecond lasers) have many applications as important tools for the investigation of ultrafast phenomena. Ultrafast laser-matter interaction may induce changes to the structure and refractive index of materials. Although it is more difficult to measure the polarization of a material in the ultrafast optical regime using a single pulse with a coherence length of 10^−6^–10^−2^ m (corresponding to a bandwidth of 10 fs–100 ps), several suitable methods have been reported. In 2006, Bellouard et al. used polarization microscopy based on off-axis digital holography [32] to measure the birefringence of fused silica exposed to femtosecond laser pulses [33]. However, the instrument can only measure and obtain the polarization data of static objects. In 2017, Yue et al. proposed a time-resolved holographic polarization microscopy method based on angular multiplexing holography to study ultrafast phenomena in birefringent transparent materials [34]. This method can contrast the amplitude and the phase changes of two orthogonal polarized orientations at two different times but cannot extract the four parameters of the Jones matrix, which can precisely describe the polarization of an optical field. Thus, a technique for using an ultrafast laser to accurately measure individual anisotropic samples and dynamic objects is needed.

In this report, we propose a novel and simple four-channel Jones matrix measurement of birefringent materials method based on ultrafast time-resolved angular multiplexing. In the proposed system, two delay lines are used to generate specific time delays between two orthogonally polarized sub-pulses. Provided that the delay time is less than the exposure time of the charge-coupled device (CCD) camera, a four-channel angular multiplexing hologram can be obtained based on a single recording. By skillfully adjusting the directions of two 2D orthogonal gratings and the polarizers of four pinholes filter, two orthogonally polarized object beams can interfere with their corresponding reference beams in the recording plane, thus forming a four-channel angular multiplexing hologram. In this method, a four-channel angular multiplexing hologram can be recorded using a single pulse. By extracting the spatial spectrum of the hologram, the Jones matrix information of the birefringent sample can be easily recovered. The feasibility of this method was experimentally demonstrated by measuring three samples. We measured the amplitude and phase distributions of the USAF 1951 resolution test target in two orthogonal polarization directions, as well as the amplitude and phase distributions of its Jones matrix parameters. The results confirmed that the proposed system has a high spatial resolution and can measure the Jones matrix of non-birefringent objects. We also measured the amplitude and phase distributions of potato starch particles in a microfluidic chip in two orthogonal polarization directions, as well as the amplitude and phase distributions of their Jones matrix parameters. This established that the proposed system can also measure the Jones matrix of dynamic birefringent objects. Finally, we measured the complex amplitudes and phase retardations of liquid crystal cell for different applied voltages (0 V, 2 V, 3 V, and 4 V), described and accounted for the observed changes, and then measured the amplitude and phase distributions of the Jones matrix parameters of liquid crystal cell for a voltage of 4 V (when voltage beyond 4 V, the distribution of the Jones matrix parameters of liquid crystals will not exhibit distinct changes).

## 2. Materials and Methods

**Figure 1:**
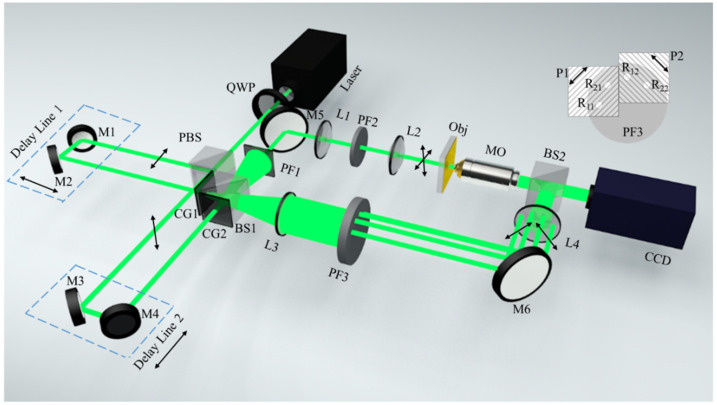
Schematic illustration of the proposed four-channel Jones matrix measurement system. QWP, quarter-wave plate; BS1–BS2, beam splitters; PBS, polarizing beam splitter; M1–M6, mirrors; P1–P2, linear polarizers; CG1–CG2, 2D orthogonal gratings; PF1–PF3, pinholes filters; L1–L4, lens; MO, microscope objectives; CCD, charge-coupled device.

The schematic of the proposed system is represented in Figure 1. The linearly polarized single pulse beam emitted from the ultrafast pulse laser (a commercial picosecond laser; Ekspla PL2250, central wavelength 532 nm, pulse duration approximately 30 ps) is first converted into circularly polarized light using a quarter-wave plate QWP, then divided into the following two detection sub-pulses with orthogonal linear polarization states using the polarizing splitter prism PBS: a first sub-pulse with horizontal polarization and a second sub-pulse with vertical polarization. The first sub-pulse is incident on the two-dimensional orthogonal grating CG1 after passing through a delay line composed of mirrors M1 and M2; the second sub-pulse is incident on the two-dimensional orthogonal grating CG2 after passing through the delay line composed of M3 and M4. The detection pulses that pass through CG1 and CG2 are divided into the following two paths after traversing through the non-polarizing beam splitter prism BS1: one as the object light and the other as the reference light. A single pinhole spatial filter PF1 is set in the object light path. The pinhole filter only allows the zero-order diffraction pulse from grating CG1 and grating CG2 to pass through; the two detection pulses with an orthogonal polarization states that through the pinhole filter PF2 irradiates the sample successively. A four-pinhole spatial filter PF3 is set in the reference optical path, which blocks the zero-order diffracted light from CG1 and CG2, whereas only four sub-pulses of the first-order diffracted terms of the two gratings are allowed to pass through. The two sub-pulses *R*_11_ and *R*_12_ are from the two orthogonal first-order diffraction terms of the orthogonal grating CG1, and the other two sub-pulses *R*_21_ and *R*_22_ are from the two orthogonal first-order diffraction terms of the orthogonal grating CG2. Assuming that the spatial orientation of grating CG2 rotates 45 degrees relative to grating CG1, the spatial distribution of the four filtering pinholes on the four-pinhole spatial filter PF3 can be set as shown in the inset of Figure 1. At the same time, the two orthogonal linear polarizers P1 and P2 are also set at the four-filter pinhole positions of the four-pinhole filter PF3. After passing through the polarizers P1 and P2, the four reference beams become two groups of orthogonal linearly polarized light. The sub-pulses *R*_11_ and *R*_21_ are linearly polarized at an angle of +45 degrees relative to the vertical direction, whereas sub-pulses *R*_12_ and *R*_22_ are linearly polarized at an angle of −45 degrees relative to the vertical direction. The two sub-pulses in the object light path arrive at the recording plane after passing through filter PF1, reflector M5, lens L1, low-pass filter PF2, lens L2, dynamic sample object Obj, microscopic objective MO, and the non-polarizing beam splitter BS2. The four sub-pulses in the reference optical path reach the recording plane after passing through lens L3, four-pinhole polarization filter PF3, polarizer P1, P2, reflector M6, lens L4, and beam splitter BS2. The four-reference beam sub-pulses and two object beam sub-pulses are superposed on the recording plane after passing through the splitting prism. The superposed light field is recorded by an externally triggered CCD camera that is synchronized with the ultrafast laser, and a four-channel angular multiplexing polarization hologram (AMPH) containing the information of each orthogonal polarization component of the object at different times is obtained.

**Figure 2:**
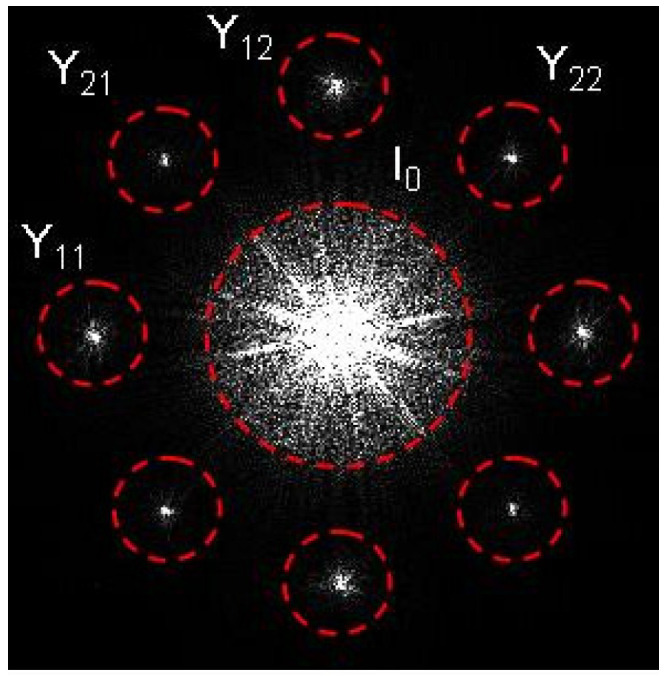
Schematic diagram of spatial spectrum distribution of the four-channel AMPH.

The time interval between the two detected pulses is adjusted so that $\Delta \tau =t_{2}-t_{1}$ (*t*_1_ and *t*_2_ are the times when two detected pulses arrive at the sample to be tested) is greater than the pulse width of the laser pulse. Each orthogonal polarization component of the two object beams corresponding to different detected pulses can only interfere with the orthogonal polarization component of the reference beam from the same detected pulse. Therefore, the total intensity *I* on the CCD recording plane can be expressed as follows:

(1) $$I={\left|E_{1}\left(\begin{array}{cc} T_{xx}^{\left(1\right)} & T_{xy}^{\left(1\right)}\\ T_{yx}^{\left(1\right)} & T_{yy}^{\left(1\right)} \end{array}\right)\left(\begin{array}{c} 1\\ 0 \end{array}\right)+R_{11}\left(\begin{array}{c} 1\\ 1 \end{array}\right)+R_{12}\left(\begin{array}{c} 1\\ -1 \end{array}\right)\right|}^{2}+{\left|E_{2}\left(\begin{array}{cc} T_{xx}^{\left(2\right)} & T_{xy}^{\left(2\right)}\\ T_{yx}^{\left(2\right)} & T_{yy}^{\left(2\right)} \end{array}\right)\left(\begin{array}{c} 0\\ 1 \end{array}\right)+R_{21}\left(\begin{array}{c} 1\\ 1 \end{array}\right)+R_{22}\left(\begin{array}{c} 1\\ -1 \end{array}\right)\right|}^{2}$$

where, *E*_1_ and *E*_2_ are the complex amplitudes of two probe sub-pulses that are successively incident on the sample to be measured, and *T_xx_*, *T_xy_*, *T_yx_*, and *T_yy_* are the four Jones vector transmission matrix parameters of the sample. Equation (1) can be rewritten as follows:

(2) $$I=I_{0}+Y_{11}+Y_{12}+Y_{21}+Y_{22}+Y_{11}^{*}+Y_{12}^{*}+Y_{21}^{*}+Y_{22}^{*}$$

where,

(3) $$I_{0}={\left|E_{1}T_{xx}^{\left(1\right)}\right|}^{2}+{\left|E_{1}T_{yx}^{\left(1\right)}\right|}^{2}+{\left|E_{2}T_{xy}^{\left(2\right)}\right|}^{2}+{\left|E_{2}T_{yy}^{\left(2\right)}\right|}^{2}+{\left|R_{11}\right|}^{2}+{\left|R_{21}\right|}^{2}+{\left|R_{12}\right|}^{2}+\left|R_{22}\right|$$

and

(4) $$\begin{array}{l} Y_{11}=E_{1}R_{11}^{*}(T_{xx}^{\left(1\right)}+T_{yx}^{\left(1\right)})\\ Y_{12}=E_{1}R_{12}^{*}(T_{xx}^{\left(1\right)}-T_{yx}^{\left(1\right)})\\ Y_{21}=E_{2}R_{21}^{*}(T_{xy}^{\left(2\right)}+T_{yy}^{\left(2\right)})\\ Y_{22}=E_{2}R_{22}^{*}(T_{xy}^{\left(2\right)}-T_{yy}^{\left(2\right)}) \end{array}$$

where the superscript star in Equations (2) and (4) indicates a complex conjugate operation. *I*_0_ is the zero-order term, and *Y*_11_, *Y*_12_, *Y*_21_, and *Y*_22_ correspond to the four first diffraction orders of the four-channel AMPH. The total intensity for Equation (2) can be recorded using an ordinary image sensor to obtain a four-channel AMPH. Figure 2 is the spatial spectrum distribution diagram of the hologram. The figure shows that the spatial frequency spectrums corresponding to items *Y*_11_, *Y*_12_, *Y*_21_, and *Y*_22_ are separated from those of the other terms in Equation (2) in the spatial frequency domain. Therefore, the spatial filtering algorithm for reconstructing off-axis holograms [35,36,37] can be used to extract them. In the retrieving procedure, we first transform the four-channel function AMPH into the spatial frequency domain using a fast Fourier transform (FFT) algorithm. The spatial spectra of the four terms in Equation (4) are then separated from the other terms in the spatial frequency domain, which allows for extraction using spatial filtering. Thus, the four wanted complex amplitudes *Y*_11_, *Y*_12_, *Y*_21_ and *Y*_22_ can be retrieved from the extracted spectra components using an inverse FFT algorithm. The Jones vector transmission matrix parameters of the sample can then be obtained using the following equations:

(5) $$\begin{array}{l} T_{xx}^{\left(1\right)}=\frac{1}{2}\left(\frac{Y_{11}}{Y_{11}^{0}}+\frac{Y_{12}}{Y_{12}^{0}}\right)\\ T_{yx}^{\left(1\right)}=\frac{1}{2}\left(\frac{Y_{11}}{Y_{11}^{0}}-\frac{Y_{12}}{Y_{12}^{0}}\right)\\ T_{xy}^{\left(2\right)}=\frac{1}{2}\left(\frac{Y_{21}}{Y_{21}^{0}}-\frac{Y_{22}}{Y_{22}^{0}}\right)\\ T_{yy}^{\left(2\right)}=\frac{1}{2}\left(\frac{Y_{21}}{Y_{21}^{0}}+\frac{Y_{22}}{Y_{22}^{0}}\right) \end{array}$$

where, $Y_{11}^{0}=E_{1}R_{11}^{*}\;,Y_{12}^{0}=E_{1}R_{12}^{*}\;,Y_{21}^{0}=E_{2}R_{21}^{*}\;,Y_{22}^{0}=-E_{2}R_{22}^{*}$; they can be extracted from the background hologram recorded without the sample.

## 3. Results and Discussion

To demonstrate the feasibility of the presented method, we selected three samples for testing. We first determined the spatial resolution of the system by imaging a USAF 1951 resolution test target, then measured the Jones matrix of potato starch particles in a microfluidic chip, in addition to the complex amplitude distribution and phase delay distribution of liquid crystal cell for different voltages. In our experiments, a commercial picosecond laser (Ekspla PL2250, central wavelength 532 nm, pulse duration approximately 30 ps) was used as the light source, and a CCD image sensor with a pixel size of 3.75 μm × 3.75 μm and a pixel number of 1280 × 960 was used to record the four-channel AMPH.

### 3.1. Characterization of the Spatial Resolution and Demonstration of the Consistency

**Figure 3:**
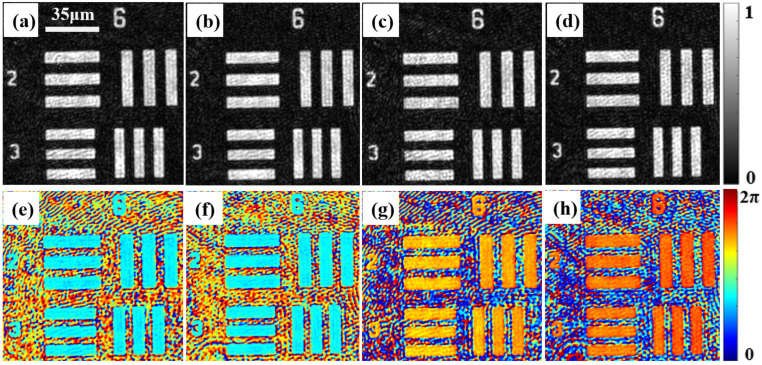
Amplitude and phase distributions of USAF 1951 resolution test target in two orthogonal polarization directions. (**a**–**d**) The amplitude distributions corresponding to *Y*_11_, *Y*_12_, *Y*_21_, and *Y*_22_, respectively; (**e**–**h**) are the corresponding phase distribution.

We chose the transmission USAF 1951 resolution test target (group 6 elements 2–3) as the first test sample. Figure 3 shows the amplitude and phase distribution extracted from the spatial spectrum of the resolution plate hologram. Figure 3a–d shows the amplitude distribution of the resolution plate, which corresponds to *Y*_11_, *Y*_12_, *Y*_21_, and *Y*_22_ in Figure 2, and Figure 3e–h shows the corresponding phase distribution. Figure 3 shows that the proposed system has a high spatial resolution and can easily resolve the smallest element (group 6, element 3) of the resolution test target. Given that the measured resolution test target is a static isotropic sample, it has the same amplitude and phase distribution in the two orthogonal directions as shown in Figure 3. 

**Figure 4:**
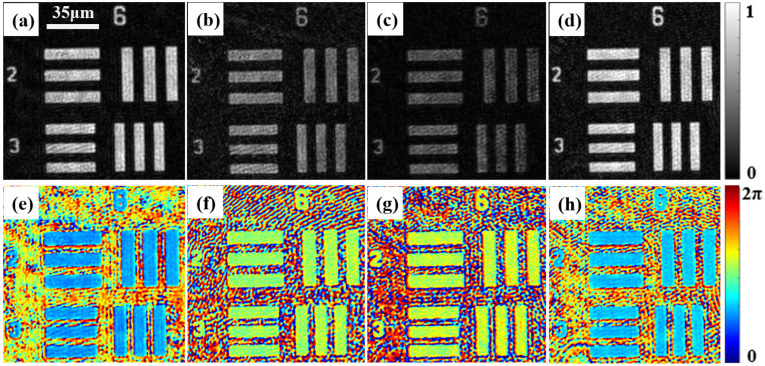
Measured Jones matrix parameters of a USAF 1951 resolution test target. (**a**–**d**) The amplitude distributions of the four Jones matrix parameters corresponding to $T_{xx}^{\left(1\right)}$, $T_{xy}^{\left(1\right)}$, $T_{yx}^{\left(2\right)}$, and $T_{yy}^{\left(2\right)}$, respectively; (**e**–**h**) are their corresponding phase distributions.

Figure 4 shows the amplitudes and phase distributions of the Jones matrix parameters of the USAF 1951 resolution test target. Figure 4a–d correspond to the amplitude distributions of $T_{xx}^{\left(1\right)}$, $T_{xy}^{\left(1\right)}$, $T_{yx}^{\left(2\right)}$ and $T_{yy}^{\left(2\right)}$, respectively, and Figure 4e–f is the corresponding phase distributions. The amplitude and phase distributions in Figure 4 were retrieved from Figure 3a–d and Figure 3e–h, respectively, using Equation (5). Given that the resolution test target is non-birefringent and a static specimen, the reconstructed amplitude distributions of *Y*_11_, *Y*_12_, *Y*_21_, and *Y*_22_ in Figure 3 are similar, and the reconstructed phase distributions have the relationship *Y*_11_ ≈ *Y*_12_ and *Y*_21_ ≈ *Y*_22_, which demonstrates that a non-birefringent material has the same amplitude and phase distributions in different directions. From Figure 4, we can determine using the relationship between the four Jones matrix parameters that $T_{xx}^{\left(1\right)}$ ≈ $T_{yy}^{\left(2\right)}$ and $T_{xy}^{\left(1\right)}$ ≈ $T_{yx}^{\left(2\right)}$, which is consistent with the theoretical prediction of Equation (5). Based on the results of the first experiment, we demonstrated that the measurement system had a high spatial resolution and was able to measure the complex amplitude distributions and even the Jones matrix components of non-birefringent material.

### 3.2. Jones Matrix Measurement of Potato Starch Granules

We also characterized the potato starch granules. They were taken from fresh potatoes and diluted in deionized water. The obtained starch granule solution was then placed in a microfluidic chip, in which the starch granules flowed.

**Figure 5:**
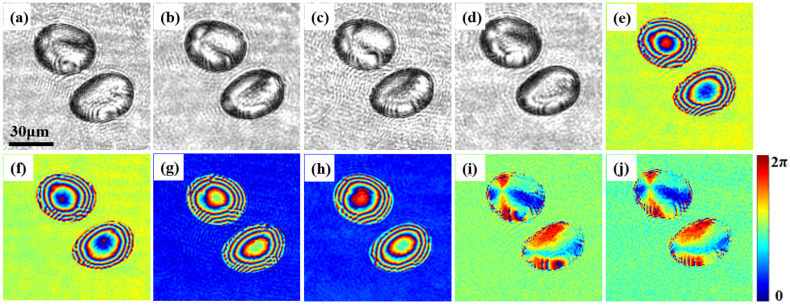
Amplitude and phase distribution of potato starch granules in two orthogonal polarization directions. (**a**–**d**) The amplitude distributions correspond to *Y*_11_, *Y*_12_, *Y*_21_, and *Y*_22_, respectively; (**e**–**h**) are the corresponding phase distributions; (**i**,**j**) are the phase delays in the two orthogonal polarization directions, respectively.

**Figure 6:**
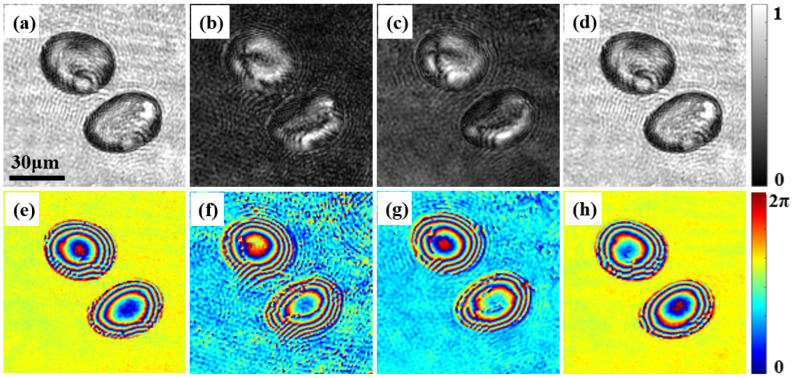
Amplitude and phase distribution of Jones matrix of potato starch granules. (**a**–**d**) The amplitude distributions correspond to $T_{xx}^{\left(1\right)}$, $T_{xy}^{\left(1\right)}$, $T_{yx}^{\left(2\right)}$, and $T_{yy}^{\left(2\right)}$, respectively; (**e**–**h**) are the corresponding phase distributions.

Figure 5 and Figure 6 show the measurement results for the potato starch granules. Figure 5a–d is the amplitude distributions extracted from the hologram, which correspond to *Y*_11_, *Y*_12_, *Y*_21_, and *Y*_22_ in the spatial spectrum. Figure 5e–h is the corresponding phase distributions. Figure 5i represents the phase delay distributions of the two phases extracted from *Y*_11_ and *Y*_12_, which is the phase difference between Figure 5e,f. Similarly, Figure 5j represents the phase difference between Figure 5g,h.

In Figure 5, the amplitude and phase distributions of the potato starch granules are different in the two orthogonal polarization directions. It can be easily shown that *Y*_11_ ≈ *Y*_22_ and *Y*_12_ ≈ *Y*_21_, but *Y*_11_ ≠ *Y*_12_. This demonstrates that birefringent material has different amplitude and phase distributions in two orthogonal polarization directions, which indicates that potato starch granules have different amplitude and phase distributions in the two orthogonal polarization directions. Figure 5i,j are nearly identical, which demonstrates that potato starch granules have the same phase delay in two orthogonal polarization directions when irradiated with light of different polarization. Moreover, the deionized water around the starch granules has the same amplitude and phase distribution in different polarization directions, which is consistent with the assumption that water is isotropic. Figure 6 shows the amplitude and phase distributions of the measured Jones matrix of potato starch granules. Figure 6a–d shows the amplitude distribution of the Jones matrix, corresponding to four Jones vectors $T_{xx}^{\left(1\right)}$, $T_{xy}^{\left(1\right)}$, $T_{yx}^{\left(2\right)}$ and $T_{yy}^{\left(2\right)}$, respectively. Figure 6e–h shows the corresponding phase distribution.

Based on careful observation of Figure 6, it can be determined that the amplitude value of the potato starch granules changes in the range of 0–1, and the phase value of the potato starch granules changes in the range of 0–2 π; whereas the amplitude and phase values of deionized water around potato starch granules are determined (the amplitude values of deionized water around potato starch granules in Figure 6a,d are the same and close to 1, and the amplitude values of deionized water around potato starch granules in Figure 6b,c are the same and close to 0; the phase values of deionized water around potato starch granules in Figure 6e,h are the same and close to π, and the phase values of deionized water around potato starch granules in Figure 6f,g are the same and close to 0). That demonstrates the difference between anisotropic and isotropic materials. From Figure 6, we can easily find the relationship between the Jones matrix parameters, i.e., $T_{xx}^{\left(1\right)}$ ≈ $T_{yy}^{\left(2\right)}$ and $T_{xy}^{\left(1\right)}$ ≈ $T_{yx}^{\left(2\right)}$, which is consistent with the theoretical prediction based on Equation (5). From the amplitude and phase distributions shown in Figure 6, it is determined that all the Jones matrix parameters exhibit distinct amplitude and phase distribution changes in the starch particle region, which reflects the non-uniformity of the refractive index of these particles; at the same time, the amplitude and phase distribution of deionized water around potato starch granules are determined, which indicates that the refractive index of deionized water is uniform.

The experimental results for the potato starch granules show that the complex amplitude distribution recovered from the anisotropic medium and the measured Jones matrix distribution are completely different from that of the isotropic medium. It is also shown that the proposed system can realize the quantitative measurement of the Jones matrix parameters of dynamic anisotropic materials in a single shot.

### 3.3. Measurement of Liquid Crystal Cell

A liquid crystal (LC) is a prototypical example of soft matter, exhibiting diverse degrees of orientation and position ordering of anisotropic molecules, and thereby exhibiting strong birefringence [38]. 

**Figure 7:**
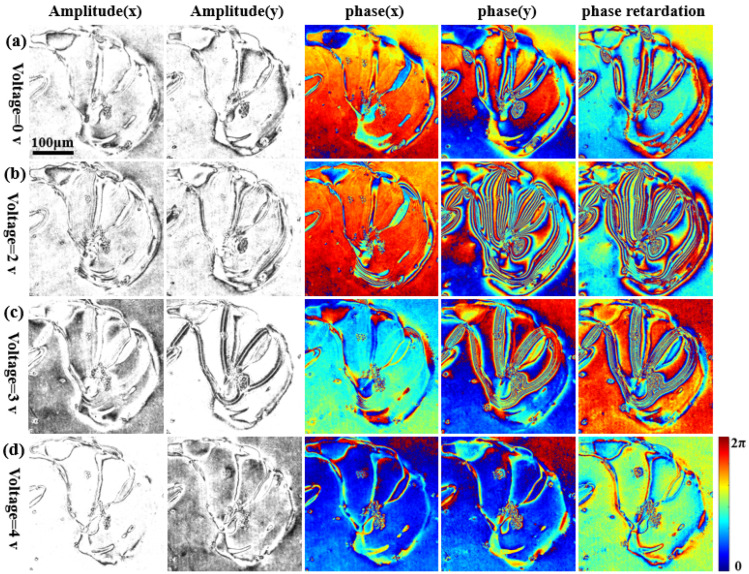
Complex amplitude distribution and phase delay distribution results for liquid crystal cell in orthogonal polarization directions for different applied voltages. (**a**–**d**) Are the measurement results for the liquid crystal cell for voltages of 0 V, 2 V, 3 V, and 4 V, respectively; from left to right, they are the amplitude distribution, phase distribution, and phase delay in the two orthogonal polarization directions.

In this experiment, different voltages were applied to the liquid crystal cell (aromatic lipid thermotropic liquid crystalline cell; the thickness of the liquid crystal cell is about 10 μm, and it is a kind of nematic liquid crystal). The liquid crystal cell under investigation was sandwiched between a glass slide and a cover glass. A DC-regulated power supply (adjustable range 0–30 V, minimum adjustment accuracy 0.1 V) was then used to apply different voltages via two wires. Figure 7 shows the measurement results. This figure shows that the phase distribution and phase delay distribution of the liquid crystal cell change distinctly for different voltages. Figure 7a shows that the phase distribution of the liquid crystal cell in the two orthogonal polarization directions is different even if a voltage is not applied. Figure 7a–d shows the changes of the complex amplitudes and phase retardations of the LC sample in electric field-free instances, and for applied voltages of 2 V, 3 V, and 4 V, respectively. Based on Figure 7a–d and the measurement results of the LC sample at different voltages, we can conclude that with the increase in the voltage, the changes in the complex amplitudes and phase retardation distributions (which reflect the birefringence) are increasingly distinct up to 2 V, as the voltage is increased further, the changes become smaller and are negligible beyond 4 V.

This is because as the voltage increases, the orientation of the LC molecules changes from disordered to ordered until the voltage approaches a threshold value for the orientations of the LC molecules, in which they are aligned in the direction of the electric field. Moreover, it is determined that with the change of the electric field, the complex amplitudes and phase retardations of the y (vertically polarized) component exhibit significant change, but the x (horizontally polarized) component exhibits little change.

**Figure 8:**
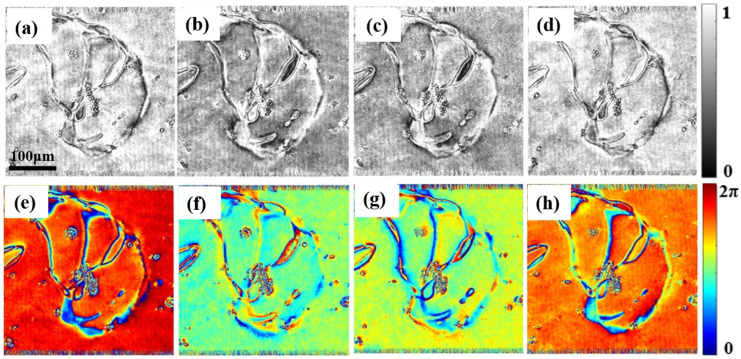
Amplitude and phase distribution of the Jones matrix of liquid crystal cell for an applied voltage of 4 V. (**a**–**d**) The amplitude distributions correspond to $T_{xx}^{\left(1\right)}$, $T_{xy}^{\left(1\right)}$, $T_{yx}^{\left(2\right)}$ and $T_{yy}^{\left(2\right)}$, respectively; (**e**–**h**) are the corresponding phase distribution.

We also measured the Jones matrix parameters of the liquid crystal cell at an applied voltage of 4 V. Figure 8 shows the amplitude and phase distributions of the measured Jones matrix of the liquid crystal cell when a voltage of 4 V is applied. Figure 8a–d shows the amplitude distribution of the Jones matrix, corresponding to the four Jones vectors $T_{xx}^{\left(1\right)}$, $T_{xy}^{\left(1\right)}$, $T_{yx}^{\left(2\right)}$ and $T_{yy}^{\left(2\right)}$, respectively. Figure 8e–h shows the corresponding phase distribution. From Figure 8, we can also find the relationship between the Jones matrix parameters given by $T_{xx}^{\left(1\right)}$ ≈ $T_{yy}^{\left(2\right)}$, and $T_{xy}^{\left(1\right)}$ ≈ $T_{yx}^{\left(2\right)}$, which is consistent with the theoretical prediction based on Equation (5). Based on the distribution of the Jones matrix of the liquid crystal cell and Figure 7, it can be determined that beyond 4 V, the LC molecules have the same orientation as that of the electric fields. Therefore, the distribution of the Jones matrix parameters will not exhibit distinct changes.

## 4. Conclusions

In summary, we proposed a novel and simple time-resolved four-channel Jones matrix measurement method of birefringent materials based on an ultrafast laser. Using this approach, the dynamic polarization measurement of microscopic objects can be realized, and the Jones matrix of birefringent materials can be quantitatively measured in a single shot. First, we determined the spatial resolution of the system by measuring the amplitude and phase distributions of a USAF 1951 resolution test target in two orthogonal polarization directions. The results confirmed that the system has a high spatial resolution. Moreover, it can measure complex amplitude distributions and even the Jones matrix components of non-birefringent material. We also measured the amplitude and phase distributions of the Jones matrix parameters of potato starch particles in a microfluidic chip. The results revealed differences in the Jones matrix components between anisotropic and isotropic materials and demonstrated that the developed instrument can realize the quantitative measurement of the Jones matrix parameters of dynamic anisotropic materials. Finally, we measured the complex amplitude distribution and phase delay distribution of liquid crystal cell under different applied voltages. The observed behavior was described, and explanations of the results were presented. We also measured the amplitude and phase distributions of the Jones matrix parameters of liquid crystal cell for an applied voltage of 4 V. In addition, if we change the picosecond laser to a femtosecond source, provided that the time interval between the two detected pulses is greater than the pulse width, it may be possible to achieve real-time measurement of the Jones matrix of ultrafast dynamic processes.

## Data Availability

Not applicable.
